# Effect of combined probiotics and doxycycline therapy on the gut–skin axis in rosacea

**DOI:** 10.1128/msystems.01201-24

**Published:** 2024-10-30

**Authors:** Jie Yu, Yan Duan, Meng Zhang, Qi Li, Miao Cao, Weixin Song, Feiyan Zhao, Lai-Yu Kwok, Heping Zhang, Ruiya Li, Zhihong Sun

**Affiliations:** 1 Key Laboratory of Dairy Biotechnology and Engineering, Ministry of Education, Inner Mongolia Agricultural University, Hohhot, China; 2 Key Laboratory of Dairy Products Processing, Ministry of Agriculture and Rural Affairs, Inner Mongolia Agricultural University, Hohhot, China; 3 Inner Mongolia Key Laboratory of Dairy Biotechnology and Engineering, Inner Mongolia Agricultural University, Hohhot, China; 4 Department of Dermatology, Inner Mongolia People’s Hospital, Hohhot, China; APC Microbiome Ireland, Cork, Ireland

**Keywords:** rosacea, probiotic, skin microbiota, gut microbiota, antibiotic resistance gene, gut-skin axis

## Abstract

Rosacea is a chronic inflammatory skin condition marked by facial erythema, telangiectasia, and acne-like eruptions, affecting millions worldwide. While antibiotics remain a common treatment, prolonged use has significant adverse effects and can lead to antibiotic resistance. This study evaluated the impact of combined probiotics and doxycycline treatment on rosacea, emphasizing the gut–skin axis. Sixty rosacea patients were randomly assigned to the probiotic, placebo, or control groups. After a 2-week doxycycline treatment, participants underwent a 3-month intervention with either a placebo, probiotic, or no further treatment. Clinical outcomes were assessed at baseline and after the 14-week intervention. Our results showed that probiotic administration improved facial skin conditions, alleviated inflammation, and reduced facial skin microbiota diversity while enhancing gut microbiota heterogeneity. Multivariate analysis identified microbial markers distinguishing the probiotic group from the control and placebo groups, and some markers were associated with skin health parameters. After the probiotic intervention, some facial skin-associated taxa, such as *Aquabacterium* sp., *UBA4096* sp. 1, *UBA4096* sp. 2, and *Yimella indica*, decreased in abundance. Additionally, the fecal microbiota of the probiotic group was enriched in specific gut microbes, including *Streptococcus parasanguinis*, *Erysipelatoclostridium ramosum*, and *Coprobacillus cateniformis*, while showing a reduced abundance of *Bacteroides vulgatus*. These changes were associated with reduced facial sebum levels and a lower physician’s global assessment score. Finally, fewer antibiotic resistance genes, particularly tetracycline resistance genes, were detected in the probiotic group compared with the control and placebo groups. Our study supports the existence of a gut–skin axis and the application of probiotics in managing rosacea.

**IMPORTANCE:**

This research elucidates rosacea management with novel insights into probiotic use alongside doxycycline, showing dual benefits in symptom relief and inflammation reduction in patients. The study maps probiotic-induced shifts in gut and skin microbiota, underscoring microbial shifts correlating with skin health improvements. Crucially, it deciphers the gut–skin axis modulation by probiotics, proposing a method to curb antibiotic resistance in rosacea therapies. This study furnishes robust evidence for probiotics in rosacea, advancing our grasp of the gut–skin relationship.

## INTRODUCTION

Rosacea and acne vulgaris are prevalent skin conditions that affect millions of people worldwide (1–3). Rosacea, characterized by facial redness, flushing, papules, and pustules, often brings considerable discomfort and embarrassment (1, 2). The global prevalence of rosacea is estimated to be around 5.46% among adults, with a prevalence of approximately 3.48% in the Chinese population (4–6). In recent years, the incidence of acne vulgaris has also been on the rise (4, 6), contributing not only to cosmetic concerns but also to psychological distress and the risk of permanent scarring (7, 8). Despite the high prevalence of rosacea, emerging research has increasingly associated it with a range of systemic complications, including gastrointestinal disorders, autoimmune diseases, and neurological conditions (3). Nevertheless, the exact pathogenesis remains elusive, and existing treatment options often provide limited efficacy and are prone to adverse side effects (9–11). The growing prevalence of acne has sparked increased interest in exploring alternative and complementary treatments, such as probiotics (12–14).

The gut microbiome, a vast and intricate ecosystem composed of trillions of microorganisms, including bacteria, viruses, and fungi (15–17), is fundamental to human health (16, 18, 19). This diverse microbial community orchestrates various physiological processes, such as digestion, nutrient absorption, immune modulation, and inhibiting pathogenic bacteria (20–23). Disturbances in the gut microbiome can lead to immune system dysregulation, increased intestinal permeability, and systemic and intestinal inflammation, contributing to conditions, such as irritable bowel syndrome, inflammatory bowel disease, and obesity (24–26). The gut microbiome also plays a significant role in skin health, particularly in conditions like rosacea, where gastrointestinal comorbidities are common (3). Gut commensal microbes play a significant role in the pathogenesis of certain dermatological disorders. Furthermore, the disruption of the skin’s external barrier can lead to an imbalance in the skin’s microecology, further exacerbating these conditions (27, 28). The gut–skin axis, a concept linking gastrointestinal and skin health, explains the pathogenesis of chronic inflammatory diseases. Skin homeostasis and allostasis are closely tied to gastrointestinal health through a complex interplay of immune, metabolic, and nervous systems (3, 29). The gut microbiome, through its bidirectional regulatory influence on host immunity, is a critical component in maintaining the delicate balance within the gut–skin axis (30).

Probiotics are live microorganisms that, when consumed in adequate amounts, confer numerous health benefits by establishing a balanced gut environment (31). These benefits extend to digestive health, immune function, and even skin health (26, 32–35), highlighting the significant role of probiotics in regulating the gut microbiota and overall human well-being (23, 36, 37). Their ability to modulate the gut microbiome has sparked interest in their potential as a treatment for rosacea (27, 38). Studies suggest that both oral and topical applications of probiotics can directly influence skin microbiota and immune responses, potentially improving skin barrier function, reducing inflammation, and addressing skin microbiome dysregulation by restoring cytokine balance (39, 40). For instance, probiotics may target toll-like receptor 2, which is often upregulated in rosacea (41). Furthermore, oral probiotics can modulate intestinal microbiota, thereby indirectly improving skin conditions (30). Although these findings are promising, further research is essential to fully establish the efficacy of probiotics in treating rosacea (3, 38).

The present study was designed to assess the efficacy of oral compound probiotics in improving facial rosacea. Sixty participants were recruited and randomly assigned to one of three groups: a control group, a placebo group, and a probiotic group. Initially, all participants received a two-week course of oral doxycycline, followed by a 3-month intervention with or without the addition of compound probiotics. The outcomes were evaluated through changes in the physician’s global assessment (PGA) score (42, 43), as well as assessments of facial skin parameters, including stratum corneum (SC) hydration, sebum level, skin pH, and microbiota composition, alongside serum cytokine levels (interleukin-8 [IL-8], kallikrein 5 [KLK5], tumor necrosis factor-alpha [TNF-α]), the serum level of an antimicrobial peptide (LL-37) levels, and fecal metagenomic analysis. Our findings indicate that oral probiotic supplementation can significantly improve rosacea symptoms, thereby reinforcing the concept of the skin–gut axis as a critical mechanism in maintaining skin health and homeostasis. This study suggests that modulating the skin–gut axis through probiotic intervention represents a promising strategy for managing rosacea.

## MATERIALS AND METHODS

### Trial design and volunteer recruitment

A randomized controlled trial was conducted at the Inner Mongolia People’s Hospital (Hohhot, China) from May to December 2019, over a period of 14 weeks. The study adhered strictly to the principles of the Helsinki Declaration and followed good clinical practice regulations. The research protocol was approved by the Ethics Committee of the Inner Mongolia People’s Hospital (Approval No. 201811004). Written informed consent was obtained from each participant before the start of the trial.

Before commencing the trial, the symptoms of facial erythema, telangiectasia, papules, pustules, and itching in recruited subjects were assessed based on the diagnostic criteria outlined in Table S1 (44, 45). Subjects with a total score >5 were diagnosed with rosacea. Patients with rosacea and who expressed their willingness to participate were further screened for their suitability to join this study based on the exclusion criteria. Patients were excluded if they (1) had severe gastrointestinal disorders or organic lesions in the gastrointestinal tract, such as inflammatory bowel disease and colon cancer; (2) received prolonged antibiotic treatment and/or probiotic intake within 1 month before the disease diagnosis; (3) had a recent history of *Clostridioides difficile* infection within the past 3 months; (4) were trying to conceive (including both males and females), pregnant, or breastfeeding; (5) had immunosuppressive diseases and/or underwent related treatment within the past year; (6) had chronic respiratory allergies and received regular anti-allergy medications; or (7) had any allergies to probiotics or any component of intervention material used in this study.

**Fig 1:**
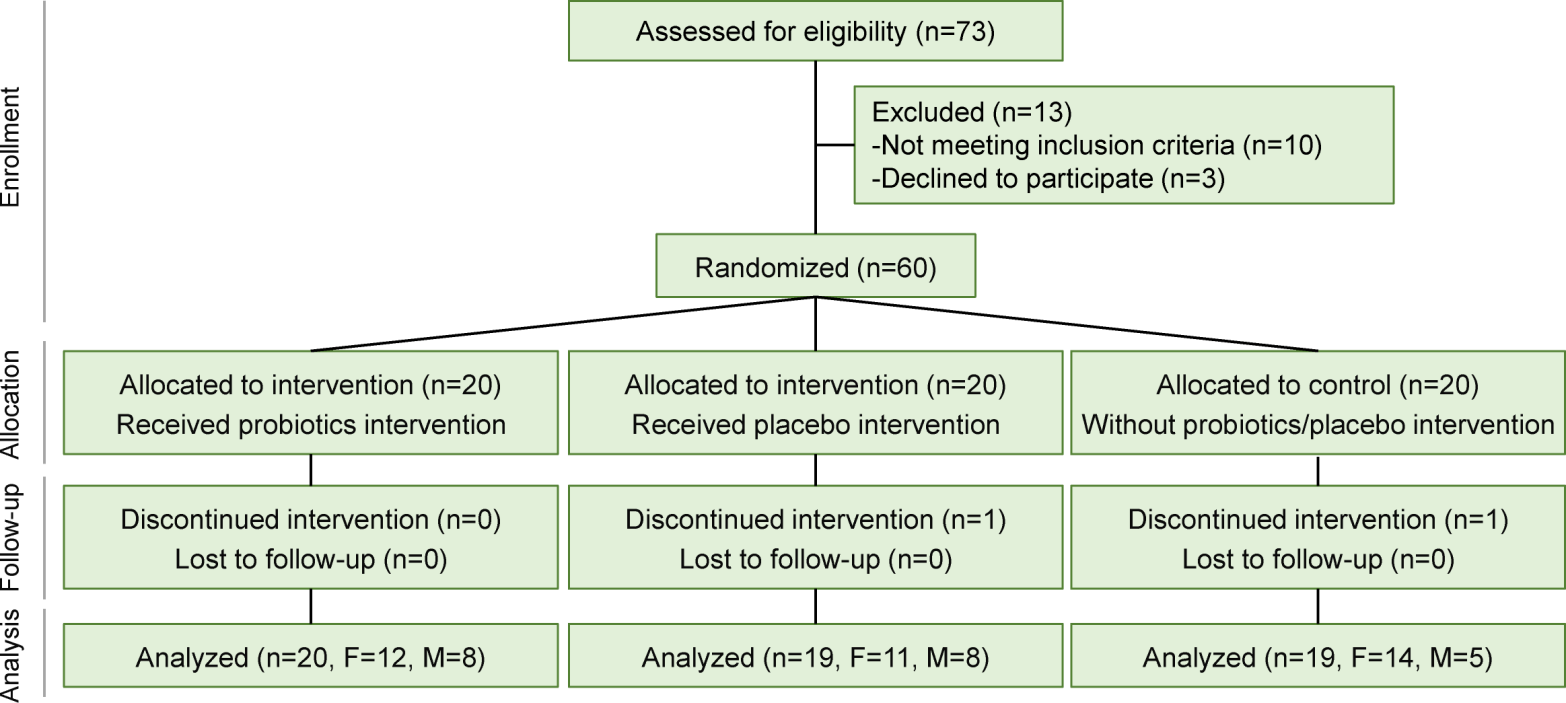
Subject flow diagram. A total of 60 patients were recruited using an intention-to-treat approach after screening based on the pre-defined inclusion and exclusion criteria. Randomization of participants was performed using R and a random number generator. A total of 58 patients were included in the per-protocol analysis after excluding one subject each from the control and placebo groups. No adverse events or serious adverse events were reported by any patient. At each follow-up, a team of physicians and scientists jointly assessed compliance and the subjects’ clinical presentations.

Following the preliminary screening, which excluded 13 patients, 60 participants (23 males and 37 females) were enrolled in the study (Fig. 1). To ensure accurate and unbiased comparisons, the participants were randomly assigned to the probiotic, placebo, or control group using the “random” package in R (v4.0.2), with 20 individuals assigned to each group. The study employed a tripartite design, in which all participants first received a 2-week doxycycline treatment hydrochloride (50 mg per administration, twice daily). Following this initial treatment, the study comprised three distinct groups: the probiotic, control, and placebo groups. The probiotic group then received the probiotic treatment (2 g of a mixed probiotic supplement daily for 3 months). The probiotic comprised 9.70 Log_10_ CFU of *Lacticaseibacillus paracasei* Zhang, 9.70 Log_10_ CFU of *Lactiplantibacillus plantarum* P-8, 9.70 Log_10_ CFU of *Lacticaseibacillus rhamnosus* Probio-M9, 9.88 Log_10_ CFU of *Bifidobacterium* (*B*.) *animalis* subsp. *lactis* V9, and 9.88 Log_10_ CFU of *B. animalis* subsp. *lactis* Probio-M8. The control group consisted of rosacea patients who received no further treatment after the initial doxycycline. The placebo group, on the other hand, received an equivalent amount of non-active placebo material, consisting of only maltodextrin, with a similar taste and appearance to the probiotic formulation for 3 months. Both the mixed probiotic and placebo products were provided by Jinhua Yinhe Biological Technology Co., Ltd. (Zhejiang, China). This tripartite design allowed for a comprehensive analysis, as it enabled the differentiation between the specific effects of the probiotic treatment, the placebo effect, and the natural progression of the disease after the initial doxycycline treatment. The tripartite approach provided a robust framework to assess the efficacy of the probiotic treatment, as it allowed for the disentanglement of the various factors that could have influenced the outcomes, thereby enhancing the validity and reliability of the study’s findings.

Throughout the study, we implemented standardized skincare and dietary regimens for participants, following the guidelines outlined in the “Chinese Rosacea Treatment Consensus (2016).” The Participants were specifically advised to use moisturizing and emollient products regularly, apply sunscreen to protect their skin from UV damage, and avoid skincare products containing alkaline substances or ethanol, as these can irritate the skin. Additionally, they were encouraged to reduce stress and manage emotional fluctuations. In terms of diet, the participants were instructed to avoid spicy and irritating foods, refrain from alcohol consumption, and limit their intake of sweets and desserts.

At the end of the trial, one participant each from the control group and the placebo group was excluded due to discontinued intervention (Fig. 1). No participants reported any adverse effect, such as dyspepsia, nausea, heartburn, vomiting, diarrhea, or constipation, resulting from probiotics ingestion.

### Clinical assessments and sample collection

Before the initiation of doxycycline hydrochloride treatment and upon completion of the intervention, the following assessments and procedures were conducted: (1) diagnosing patients’ severity of rosacea based on the PGA score criteria (42, 43); (2) evaluating participants’ skin conditions based on SC hydration, sebum levels, and pH value; (3) capturing facial lesion images with the VISIA skin imaging system; and (4) collecting whole blood, facial rosacea content, and fecal samples.

The whole blood samples were centrifuged at 3,000 rpm for 15 min within 1 h of collection to obtain serum samples, which were stored at −80°C until further analysis. The serum levels of several inflammatory cytokines (namely IL-8, KLK5, TNF-α) and the antimicrobial peptide (LL-37) were quantified using enzyme-linked immunosorbent assay kits (Thermo Fisher Scientific Inc., Waltham, MA, USA).

Facial skin tissue samples were collected using sterile swabs by a qualified healthcare professional when collecting whole blood samples at the clinical visits. Fecal samples were self-collected by the participants using the Longsee stool storage kit (Guangdong Longsee Biomedical Co., Ltd, Guangzhou, Guangdong, China). All facial skin tissue samples and fecal samples were stored in dry ice separately, transported back to Inner Mongolia Agricultural University at the end of each day’s consultation, and kept at −80°C freezer before 16S rRNA sequencing and metagenomic sequencing, respectively.

### DNA extraction, 16S rRNA sequencing, and facial skin microbiota analysis

Genomic DNA from facial tissues was extracted using the Qiagen DNA Stool Mini Kit (Qiagen, Hilden, Germany) according to the manufacturer’s instructions. The extracted DNA was assessed by 0.8% agarose gel electrophoresis and spectrophotometry (NanoDrop 1000, Thermo Fisher Scientific Inc., Waltham, MA, USA).

Full-length bacterial 16S rRNA genes were amplified from the extracted DNA by polymerase chain reaction (PCR) using universal forward primer 27F (5′-GAGTTTGATCCTGGCTCAG-3′) and reverse primer 1492R (5′-ACCTTGTTACGACTT-3′) with 16 nucleotide barcodes (46). The KAPA HiFi system and HotStart DNA Polymerase (Kapa Biosystems, Inc., Wilmington, MA, USA) were used for the PCR.

The PCR cycling conditions were as follows: 95°C for 2 min; 30 cycles at 95°C for 30 s, 55°C for 30 s, and 72°C for 30 s; followed by a final extension of 72°C for 5 min (46). The quality of the PCR products was assessed using the Agilent DNA 1000 Kit and Agilent 2100 Bioanalyzer (Agilent Technologies Inc., Santa Clara, CA, USA). DNA libraries were prepared using the Pacific Biosciences Template Prep Kit 2.0 (PacBio Biosciences, Inc., Menlo Park, CA, USA), followed by sequencing on a PacBio RS II instrument equipped with P6-C4 chemistry (46). Raw data analysis was conducted utilizing the RS_ReadsOfInsert.1 protocol of the SMRT Portal version 2.7 (PacBio Biosciences, Inc., Menlo Park, CA, USA). Restrictive filtering parameters were implemented: a minimum of five complete passes, a minimum predicted accuracy of 90%, and defined minimum and maximum read lengths of inserts (1,400 and 1,800 bp, respectively).

Raw sequencing reads were demultiplexed and quality filtered using the standard procedures of QIIME 2 (47), version 2021.4) with default parameters (47, 48). The QIIME2 plugin (version 2021.4.0) was used to denoise, de-replicate, and count amplicon sequence variants (ASVs) using the following parameters: (1) forward and reverse reads were truncated to 1,300 bases; (2) forward and reverse reads with the number of expected errors higher than 2.0 were discarded; and (3) chimeras were detected using the “consensus” method and removed. Taxonomies were assigned to final sequences using the Silva database (v138, nr99) and the classify-sklearn procedure (49). Chloroplastic and mitochondrial ASVs were removed from the data set.

To achieve more accurate taxonomic annotation of the representative ASVs, we downloaded 5,779 human skin bacterial genomes (Unified Human Skin Genome, UHSG) from a previous study (50). From this data set, we selected 2,111 high-quality genomes (with ≥80% completeness and ≤5% contamination as assessed by CheckM [51]) to construct a reference database (23). The representative sequences obtained from QIIME2 were then annotated using BLAST against this custom database (52). Since the original genomes did not include species-level classifications, we further annotated these genomes using the latest version of the Genome Taxonomy Database (GTDB, r220) (53) *via* the GTDB-Tk (54) tool, ensuring precise and comprehensive taxonomic classification.

### Shotgun metagenomic sequencing and fecal metagenome analysis

Fecal metagenomic DNA was extracted from the fecal samples using the Magnetic Soil and Stool DNA Kit (Tiangen Biotech Co., Ltd., Beijing, China) following the manufacturer’s instructions. This kit is specifically designed to efficiently isolate high-quality DNA from complex microbial communities present in fecal samples. The NEBNext Ultra DNA Library Prep Kit for Illumina (New England Biolabs, Ipswich, MA, USA) was employed to prepare the DNA libraries for sequencing. This kit incorporates advanced enzymatic processes to fragment the DNA and add adaptors required for the subsequent sequencing process. The library preparation step ensures the generation of representative and unbiased DNA fragments for sequencing. The fecal metagenomes were sequenced using the Illumina NovaSeq 6000 platform, generating paired-end reads of 150 bp in both forward and reverse directions.

To ensure the quality of the sequencing data, a data-filtering step was incorporated using the KneadData pipeline (https://github.com/biobakery/kneaddata). This step removed low-quality reads and eliminated any human host-contaminating sequences that might be present in the data set. By employing stringent quality control measures, the resulting data set was anticipated to consist of high-quality clean reads suitable for downstream analysis. On average, each sample yielded approximately 37,344,057 high-quality paired-end clean reads.

Comprehensive metagenomic data analysis, including read assembly and contig binning, was conducted. The metagenomic reads were initially assembled into contigs using an advanced genome assembler, MEGAHIT (55), to generate longer contigs. Specifically, contigs exceeding 2,000 bp were selected for binning using two robust binning tools, MetaBAT2 (56) and VAMB (57). The binning outputs from these tools were then integrated using the powerful MetaWRAP software, to facilitate metagenome-assembled genome extraction. Subsequently, the original reads from the samples were mapped back to the corresponding contigs using BWA-MEM2 (58), and the contig depth was determined using the jgj_summarize_bam_contig_depths function in MetaBAT2 (56). The quality assessment of metagenome-assembled genomes was conducted using CheckM (51), which categorized them into high-quality levels based on a predetermined threshold of ≥80% completeness and ≤5% contamination. To further refine the analysis, the high-quality metagenome-assembled genomes underwent clustering, and representative genomes from each replicate set were selected using dRep, employing an average nucleotide identity threshold of 95%. These representative genomes, referred to as species-level genome bins, were subsequently subjected to annotation using Genome Database Taxonomy (GTDB)-Tk with the GTDB (r202) as the reference database. The relative taxonomic abundance (59) of species-level genome bins was converted to reads per kilobase per million using the CoverM software (https://github.com/wwood/CoverM), enabling quantitative assessment of representative genomes in the metagenomic data set. Lastly, the merged binners were employed for the annotation of antibiotic resistance genes (ARGs) in the metagenomic data set using PathoFact v1.0 (expressed in reads per kilobase per million, RPKM)(60).

### Statistical analysis

To conduct comprehensive statistical analyses of the microbiota and ARGs, we employed cutting-edge bioinformatics tools and methodologies. Initially, we utilized the advanced software Parallel-Meta 3.5 to calculate and visualize key diversity metrics, such as the Shannon index for alpha diversity and principal coordinate analyses (PCoA) for beta diversity. The dissimilarity between samples, measured by the Bray–Curtis dissimilarity index, was evaluated using non-parametric multivariate analysis of variance (PERMANOVA, 999 random permutations), implemented through the “vegan” package in R. Physiological and biochemical indicators as well as ASV abundance variations were analyzed using Wilcoxon rank-sum tests.

During the probiotic intervention, microbial biomarkers were identified using Microbiome Multivariable Association with Linear Model 2 (MaAsLin2) (61). We conducted pairwise comparisons between the probiotic, placebo, and control groups, identifying significantly differential facial microbiota, gut microbiota, and functional microbiota (*P* < 0.05) after the intervention (61). To assess the overall correlation between distance matrices of facial skin microbiome markers and gut bacterial communities, the Mantel test was applied using the “linkET” package in R. This approach offered significant advantages by allowing for the evaluation of complex relationships between two datasets, taking into account the multidimensional structure of the data. By focusing on distance matrices, the Mantel test provided a robust measure of similarity that effectively captured the inter-sample relationships and overall patterns within the data sets.

Significantly differential ARGs were determined using one-tailed *t*-tests, performed with R. Specifically, paired *t*-tests were employed to compare paired samples within each group across two time points from the same individual. Similarly, unpaired *t*-tests were utilized to compare samples between two groups at a specific time point.

Data visualization in this study was achieved using R, including the “ggplot2” and “ggpubr” packages, for generating boxplots, scatter plots, and line charts, and “pheatmap” for producing the heatmaps.

STORMS checklist is available at 10.6084/m9.figshare.25507009.

## RESULTS

### Participants’ baseline characteristics

To evaluate the relieving effects of oral multi-strain probiotic supplementation in combination with doxycycline on rosacea patients and the potential beneficial mechanisms, we recruited 60 individuals with rosacea from the Inner Mongolia People’s Hospital for a 14-week randomized, double-blind clinical trial. The participants were randomly assigned to the probiotic group (*n* = 20), placebo group (*n* = 20), or control group (*n* = 20). The mean age of the participants in the control, probiotic, and placebo groups was 48.15 ± 12.66, 42.40 ± 12.96, and 42.00 ± 13.39 years, respectively. The female-to-male ratio was 14:6, 12:8, and 12:8 in the control, probiotic, and placebo groups, respectively. The mean body mass index (BMI) was 23.21 ± 3.14, 23.24 ± 3.33, and 23.98 ± 2.33 for the control, probiotic, and placebo groups, respectively. At the start of the intervention, there were no significant differences in age, gender, or BMI among the three groups (one-way ANOVA, *P* = 0.273 for weight and *P* = 0.573 for BMI; χ test, *P* = 0.751 for gender). By the end of the probiotic intervention, one participant from each of the control and placebo groups was excluded due to discontinued intervention (Fig. 1). Thus, a total of 58 participants (control group: *n* = 19, probiotic group: *n* = 20, placebo group: *n* = 19) were included in the final analysis.

Data were collected from the clinical assessments (PGA score and skin features including SC hydration sebum levels, and skin pH) of the 58 participants at baseline and the end of the intervention. In total, 116 whole blood, 108 facial swabs, and 116 fecal samples were collected at the same time points. The whole blood samples were used to monitor changes in the levels of cytokines (including IL-8, KLK5, and TNF-α) and LL-37 using enzyme-linked immunosorbent assay kits. Facial skin microbiota and fecal metagenome analyses were performed using the facial swab and stool samples, respectively.

### Effects of probiotic intervention on facial skin features, serum cytokine, and LL-37 levels

**Fig 2:**
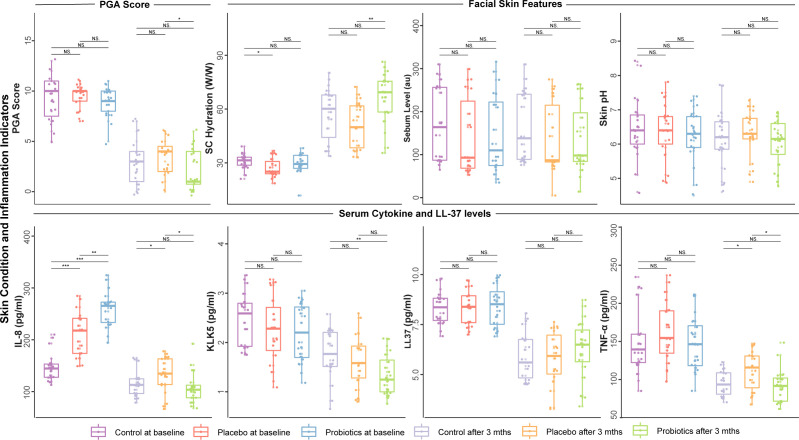
Effects of probiotic intervention on the physician’s global assessment (PGA) score, facial skin features, serum cytokine, and LL-37 levels. Boxplots present the values of various assessed parameters, including the PGA score, facial skin features (stratum corneum [SC] hydration, sebum level, skin pH), and the serum levels of the antimicrobial peptide LL-37 and cytokines (interleukin-8 [IL-8], kallikrein 5 [KLK5], tumor necrosis factor-alpha [TNF-α]). Different color bars represent the specific sample group and time point, respectively (the control group, shown in purple and light purple; the placebo group, shown in orange and light orange; and the probiotic group, shown in blue and green; at baseline and after the 3-month intervention). *P < 0.05, **P < 0.01, ***P < 0.001; NS means non-significant.

To investigate the impact of the probiotic intervention on facial skin condition and inflammatory state, we compared the monitored facial skin parameters and serum cytokine and LL-37 levels between the three sample groups at the beginning and end of the intervention. At baseline, no significant differences were observed among the three groups in PGA score, facial skin feature indicators, and serum cytokine and LL-37 levels (except for IL-8; Wilcoxon rank-sum test, *P* > 0.05; Fig. 2). However, at baseline, the probiotic group had a significantly higher level of IL-8 compared with the placebo and control groups (*P* = 0.002 and *P* = 2e-10, respectively), and the placebo group also showed a significantly higher level of IL-8 compared with the control group (*P* = 2e-05; Fig. 2).

After a 2-week doxycycline treatment and the 3-month probiotic intervention, the probiotic group had significantly lower levels of PGA score and TNF-α compared with the placebo group (*P* = 0.019 and 0.026, respectively); meanwhile, significant improvement was also observed in SC hydration compared with the placebo group (*P* = 0.001; Fig. 2). Additionally, the probiotic group had significantly lower KLK5 levels compared with the control group (*P* = 0.007; Fig. 2). Interestingly, the serum IL-8 level of the probiotic group decreased to a comparable or even lower level than that of the placebo and control groups, despite its originally higher baseline level (Fig. 2).

In summary, the probiotic intervention significantly reduced PGA scores, reduced inflammatory markers IL-8, and TNF-α, and improved skin hydration compared with the placebo group, demonstrating its significant impact beyond the placebo and control groups.

### Impact of probiotic intervention on the species diversity and composition of facial and gut microbiota

Recognizing the established gut–skin axis, which plays a crucial role in maintaining skin health through the interaction of gut and skin microbiota (30), we investigated the diversity of both facial and gut microbiota at baseline and after the intervention. This approach allowed us to evaluate the systemic effects of probiotics on gut microbiota, as well as their localized impact on the facial microbiome. Analyzing the facial microbiota is vital for understanding how oral probiotics may influence skin conditions such as rosacea. This investigation offers valuable insights into the mechanisms that contribute to clinical improvements in rosacea management.

**Fig 3:**
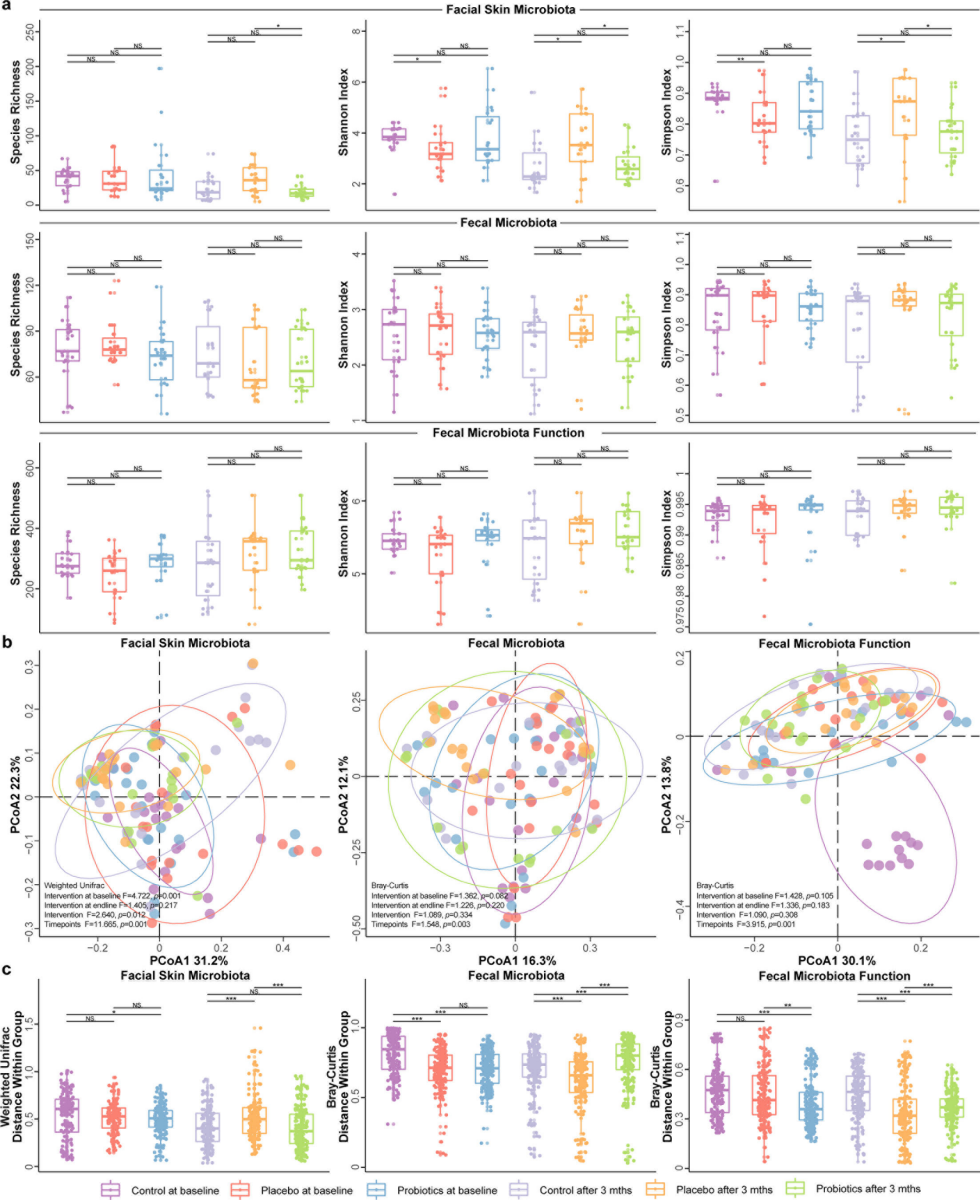
Effects of probiotic intervention on the alpha and beta diversities of the facial skin microbiota and fecal microbial metagenome. (**a**) Alpha diversity analyses (species richness, Shannon index, and Simpson’s diversity index), (**b**) principal coordinate analysis (PCoA) using UNIFRAC or Bray–Curtis distance, and (**c**) intragroup beta diversity analysis (UNIFRAC or Bray–Curtis distance) of the facial skin microbiota, fecal microbiota, and functional fecal metagenome of the control, placebo, and probiotics groups at baseline and after intervention. In the PCoA score plots, the results of PERMANOVA tests are shown, suggesting that the time factor had a significant impact on patients’ gut microbiota and functional metagenome. Different color bars represent the specific sample group and time point, respectively (the control group, shown in purple and light purple; the placebo group, shown in orange and light orange; and the probiotic group, shown in blue and green; at baseline and after the 3-month intervention). *P < 0.05, **P < 0.01, ***P < 0.001; NS means non-significant.

To investigate the inter-group differences in alpha diversity among patients, we computed species richness and both Shannon and Simpson’s diversity indices for the facial skin and fecal microbiota (Fig. 3a). At baseline, the Shannon and Simpson’s diversity indices for the facial skin microbiota in the control group were significantly higher than those in the placebo group (*P* = 0.024 and 0.006, respectively), while species richness showed no significant difference. No significant differences were observed between the probiotic group and either the control or placebo groups (*P* > 0.05). At the end of the intervention, both the Shannon and Simpson diversities of the facial skin microbiota in the probiotic and control groups were significantly lower than in the placebo group (probiotic versus placebo: *P* = 0.025 and 0.035; control versus placebo: *P* = 0.049 and 0.041, respectively). Additionally, the probiotic group exhibited significantly reduced species richness compared with the control and placebo groups (*P* = 0.01). However, no significant differences were observed in any of the calculated alpha diversity indices of gut microbiota and functional metagenome between groups (*P* > 0.05). These results suggest that both compound probiotic consumption and the absence of intervention led to reduction in the alpha diversity of the facial skin microbiota. Notably, probiotic intervention further decreased the species richness of the facial skin microbiota compared with the no-treatment group. Moreover, the facial skin microbiota exhibited a greater response than the gut microbiota in alpha diversity changes.

To assess and visualize the effect of probiotic application on the beta diversity of the facial microbiota, fecal microbiota and functional metagenome, PCoA and PERMANOVA were performed (Fig. 3b). Our analyses revealed that the overall structure of patients’ facial microbiota was significantly influenced by the time factor after the 3-month probiotic intervention (intervention: *F* = 11.665, *P* = 0.001; timepoints: *F* = 2.640, *P* = 0.012). Notably, there were baseline differences in the facial microbiota structure between groups (*F* = 4.722, *P* = 0.001), but such differences were not detected after the intervention (*F* = 1.405, *P* = 0.217; Fig. 3b). Our analyses found no significant differences in the overall structure of the fecal microbiota and functional metagenome between groups at baseline (PERMANOVA test, species: *F* = 1.362, *P* = 0.082; gene families: *F* = 1.428, *P* = 0.105) and at the end of the intervention (species: *F* = 1.226, *P* = 0.220; gene families: *F* = 1.366, *P* = 0.183). However, the time factor had a more pronounced impact on the subjects’ gut microbiota and functional metagenome (intervention: *F* = 1.089 and 1.090, *P* = 0.334 and 0.308, respectively; timepoints: *F* = 1.548 and 3.915, *P* = 0.003 and 0.001, respectively).

To further investigate the fine changes in these microbial communities, we comparatively analyzed the intra-group Bray–Curtis dissimilarity for the facial skin microbiota, fecal microbiota, and functional metagenome across the three sample groups at baseline and following the probiotic intervention. Interestingly, we observed divergent responses among the facial microbiota, fecal microbiota, and functional metagenome after the intervention period. Our results showed that both the probiotic and control groups had significantly lower intra-group Bray–Curtis dissimilarity in the facial microbiota than the placebo group (*P* = 6.1e-4 and 1.0e-5, respectively). Conversely, these groups showed significantly higher Bray–Curtis dissimilarity in the fecal microbiota and functional metagenome compared with the placebo group. Specifically, at the species level, the dissimilarity was significant for the probiotic and control groups (*P* = 1.6e-9 and 4.4e-4, respectively), and at the gene family level, the differences were also substantial (*P* = 2.8e-5 and 3.0e-13, respectively; Fig. 3c).

These results underscore the complex dynamics of microbial community responses to the probiotic intervention, highlighting the distinct effects on facial microbiota, while both the probiotic and control groups exhibited significant alterations in fecal microbiota and functional metagenomes compared with the placebo group. Overall, the findings suggest that the probiotic intervention, alongside the control treatment, can significantly influence the microbial community stability in the facial skin while affecting the diversity in fecal microbiota and functional metagenomes.

### Microbial signatures of facial and gut samples

The MaAsLin2 tool, an advanced multivariate analysis software, was employed to unveil intricate connections between the host microbiota and rosacea-related physiological characteristics while eliminating confounding factors, such as age, gender, and BMI (61).

**Fig 4:**
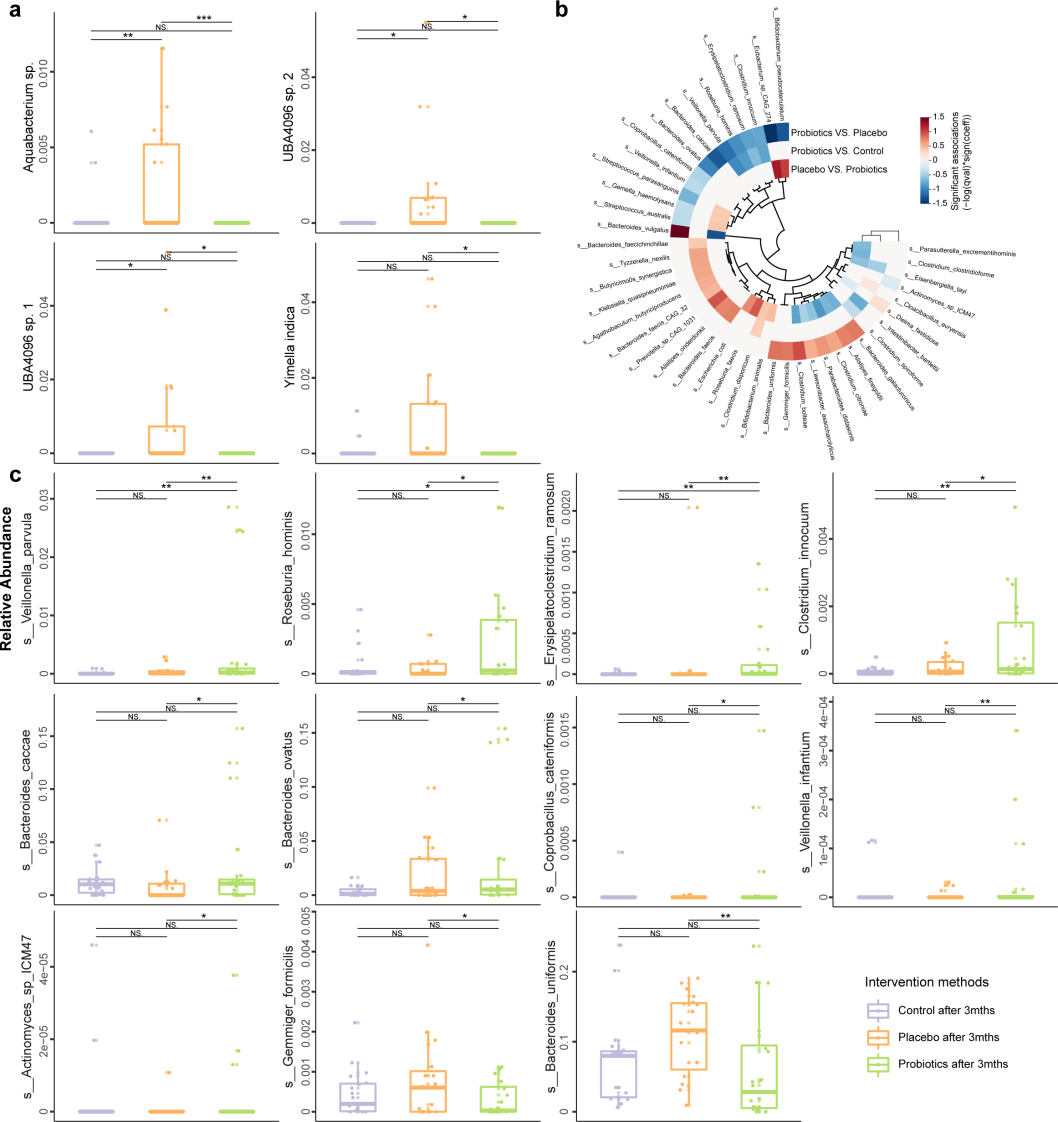
Differential species in facial skin and fecal microbiota after 3-month probiotic intervention. (**a**) Boxplots and (**b**) cladogram present the abundances of differentially abundant facial skin species and significant association coefficients (represented by the color scale) between the control, placebo, and probiotic groups, respectively. The results were generated using the MaAsLin2. (**c**) Boxplots display the abundances of differentially abundant fecal species between groups. * *P* < 0.05, **P < 0.01, ***P < 0.001; NS means non-significant.

To identify rosacea-related microbial biomarkers, we compared the facial and gut microbiota profiles between the probiotic, control, and placebo groups at baseline and at the end of the intervention (Fig. 4). This analysis identified eight responsive facial skin-associated species that showed significant changes in abundance after the probiotic intervention (*P* < 0.05; Table S2). Additionally, several significant differentially abundant facial taxa were identified between the probiotic and control/placebo groups, including *Aquabacterium* sp., *Yimella indica*, *UBA4096* sp. 1, and *UBA4096* sp. 2 (*P* < 0.05; Fig. 4a).

At the end of the intervention, 43 gut microbial species showed significant differences across the three sample groups (*P* < 0.05; Fig. 4b; Table S2). Among these, 38 species in the probiotic group exhibited significant differences when compared to the placebo and/or control groups. Specifically, four species demonstrated significant changes when compared to both the placebo and control groups, while 20 species were significantly different only from the placebo group. Additionally, 14 species showed significant differences exclusively from the control group. Furthermore, five species displayed significant differences solely between the placebo and control groups. Notably, after 3 months of probiotic intervention, the fecal microbiota of the probiotic group had significantly more *Veillonella* (*V*.) *parvula*, *Roseburia hominis*, (*Er*.) *ramosum*, and *Clostridium* (*C*.) *innocuum* than the placebo and control groups (*P* < 0.05; Fig. 4c); significantly more *Bacteroides* (*Ba*.) *caccae*, *Ba. ovatus*, *Coprobacillus* (*Cop*.) *cateniformis*, *V. infantium*, and *Actinomyces* (*A*.) sp. ICM47 than the placebo group; but significantly fewer *Gemmiger formicilis* and *Ba. uniformis* than the placebo group (*P* < 0.05; Fig. 4c). These findings suggested, although no drastic changes were observed in subjects’ facial skin and gut microbiota, the abundance of certain biomarker species did undergo substantial changes during the probiotic intervention.

### Interconnectedness among the gut microbiota, facial microbiota, and physiological parameters

**Fig 5:**
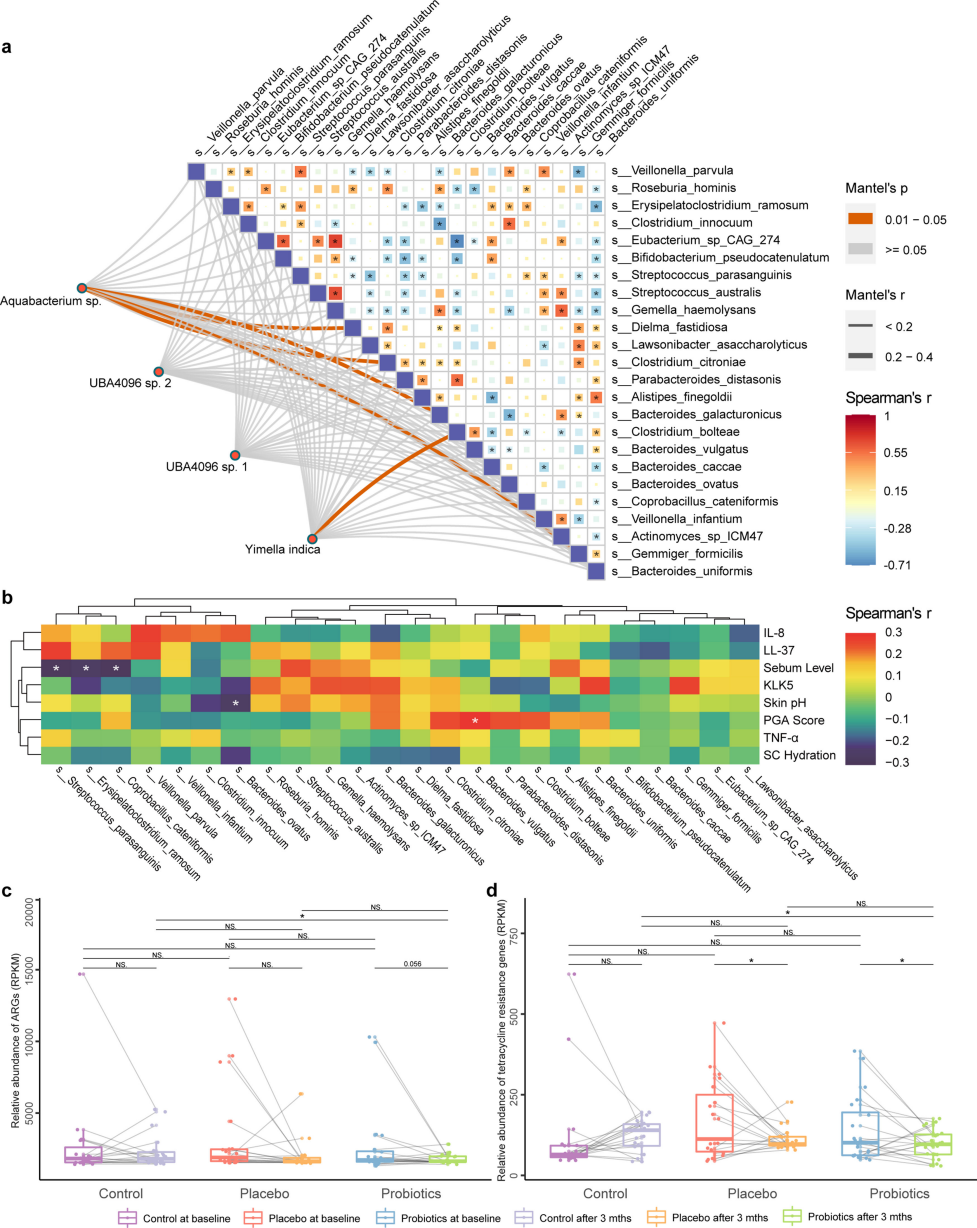
Differential microbiota, rosacea-related indicators, and antibiotic resistance genes (ARGs) in the fecal microbiota. (**a**) Correlation network between the differential facial microbiota and fecal microbiota species. The squares represent Spearman’s correlations between differentially abundant fecal species (cut-off level: *P* < 0.05; the color scale shows Spearman’s rho). The lines represent correlations between differentially abundant facial microbes and fecal microbes (determined by the Mantel test; orange and gray lines represent significant and non-significant correlations, respectively, cut-off level: *P* < 0.05; the line thickness represents the *r* value generated in the Mantel test). (**b**) Spearman’s correlations between differential gut microbiota species and rosacea-related indicators (*P < 0.05; the color scale represents Spearman’s rho). Boxplots present the gene abundance of (**c**) total ARGs and (**d**) tetracycline resistance genes in the fecal metagenome of the control, placebo, and probiotic groups. The gray lines connect the data points of the same individual at baseline and 3 months after the intervention. *P < 0.05; NS means non-significant.

To investigate the relationship between the gut microbiota, facial microbiota, and rosacea-related parameters, we conducted Spearman correlation analyses using our data sets. This analysis focused specifically on the 24 species that showed significant differences in the probiotic group compared with the placebo group, identifying key gut-responsive microbial species (Fig. 5a). Strong positive correlations were detected between *Gemella haemolysans* and *Eubacterium* (*Eu*.) sp. CAG 274, *Streptococcus* (*S*.) *australis*, and *A. sp*. ICM47 (*r* = 0.722, 0.700, 0.606; *P* = 3.12e-9, 6.16e-10, 3.86e-7, respectively; Table S3); as well as between *Eu. sp*. CAG 274 and *B. pseudocatenulatum* (*r* = 0.633, *P* = 1.77e-8; Table S3). Strong negative correlations were also found between *Eu. sp*. CAG 274 and *C. bolteae* (*r* = −0.705, *P* = 3.08e-10; Table S3), and between *C. innocuum* and *Ba. galacturonicus* (*r =* −0.623, *P* = 1.12e-07; Table S3). Some facial skin-associated microbes showed significant correlations with the gut microbiota, such as between ASV_026_DSEG and *Ba. galacturonicus*, *Gemmiger formicilis*, *Dielma fastidiosa*, and *C. citroniae* (*r* > 0.2, *P* < 0.05), and between ASV_085_DSEG and *C. bolteae* (*r* > 0.2, *P* < 0.05).

Moreover, we observed a significant positive correlation between *Ba. vulgatus* and the PGA score (*r =* 0.278, *P* = 0.035; Fig. 5b; Table S4), while the probiotic group had significantly fewer *Ba. vulgatus* than the placebo group (*P* < 0.05; Fig. 5b). The species, *S. parasanguinis*, *Er. ramosum*, and *Cop. cateniformis*, exhibited significant negative correlations with the facial sebum level (*r =* −0.32,–0.267, and −0.293, *P* = 0.021, 0.043, and 0.026, respectively; Fig. 5b; Table S4). Meanwhile, at the end of the intervention, significantly more *S. parasanguinis*, *Er. ramosum*, and *Cop. cateniformis* were identified in the probiotics group than in the placebo group (*P* < 0.05), and more *Er. ramosum* was also detected in the probiotic group than in the control group (*P* < 0.05).

In conclusion, our findings indicate that a 3-month probiotic intervention could modulate the gut microbiota, thus improving the facial microbiota and the skin conditions of the participants.

### Probiotic application decreased the abundance of ARGs in the fecal microbiota

Probiotic supplementation has been found to impact the human gut ARG reservoir in a person-specific and antibiotic-dependent manner (62, 63). In this study, all patients underwent a 2-week doxycycline treatment before different interventions. Thus, to investigate whether the probiotic intake had any effect on the intestinal ARG levels, we compared the fecal ARG levels in the three groups of subjects at baseline and after the intervention.

After the 3-month intervention, we observed an interesting trend in the fecal metagenome regarding ARGs. Both the probiotic and placebo groups exhibited reductions in ARGs, with changes approaching statistical significance (probiotic group: *P* = 0.056; placebo group: *P* = 0.060; Fig. 5c). Additionally, the probiotic group showed a significantly lower abundance of ARGs compared with the control group (*P* = 0.042), while no significant difference was detected in the placebo group (*P* = 0.215). Furthermore, when specifically analyzing tetracycline resistance genes, both the probiotic and placebo groups demonstrated significant reductions in these genes (probiotic group: *P* = 0.028; placebo group: *P* = 0.036; Fig. 5d). In contrast, the control group exhibited a non-significant increase in these genes (*P* = 0.417). Notably, the probiotic group also exhibited a significantly lower abundance of tetracycline resistance genes compared with the control group (*P* = 0.041), while the placebo group did not show a similar difference (*P* = 0.205). These findings suggest that both the probiotic and placebo interventions led to a reduction in ARGs; however, the probiotic intervention yielded a more pronounced effect, particularly when compared to the control group.

## DISCUSSION

In this study, we investigated the effect of a 3-month probiotic intervention in combination with doxycycline in managing rosacea. Specifically, we comparatively analyzed the changes in the subjects’ fecal metagenome, facial skin microbiota, and physiological indicators after the intervention. Our findings revealed significant associations between these parameters, shedding light on the potential mechanisms through which probiotics may alleviate rosacea.

The design of our study depended heavily on the baseline characteristics of the participants to ensure the randomization and comparability of the three groups. The validity of our findings was strengthened by the absence of significant differences in age, gender, or BMI between groups at baseline. The probiotic intervention exerted a symptom-relieving effect on rosacea, as evidenced by significantly lowered PGA scores, reduced TNF-α levels, and improved SC hydration. Furthermore, the decreases in KLK5 and IL-8 in the probiotic group suggested that probiotic administration could alleviate skin inflammation.

The diversity of facial microbiota is closely related to an individual’s skin health (4, 34, 64, 65). Previous studies have identified associations between certain facial conditions, such as acne, eczema, and dermatitis, and an imbalance in facial microbiota (3, 27). A reduction in diversity or an imbalance within the facial microbiota can contribute to the onset or worsening of skin problems (34, 64). Conversely, gut microbiota plays a crucial role in regulating the immune system, and a well-balanced gut microbiota is essential for maintaining normal immune function, which is closely connected to facial skin health (3). Previous studies have reported that an imbalance in gut microbiota can trigger inflammatory responses, thereby impacting facial health through the gut–skin axis (13, 15, 66). Thus, in this study, we monitored changes in both gut and facial skin microbiota of subjects following the probiotic intervention.

Notably, we observed marked alterations in the diversity of the subjects’ facial skin microbiota, as well as changes in the composition of their fecal microbiota post-intervention. Specifically, the probiotic group exhibited a significant reduction in species richness within the facial skin microbiota, while no notable changes were detected in the alpha diversity of the fecal microbiota. These findings suggest that the probiotic intervention may facilitate systemic improvements that are reflected in modulation of the facial skin microbiota. Multivariate analysis of microbial biomarkers revealed significant changes in the composition of both facial and gut microbiota at a finer taxonomic level. Specifically, after the probiotic intervention, the abundance of *Aquabacterium* sp., *UBA4096* sp. 1, *UBA4096* sp. 2, and *Yimella indica* in the facial skin microbiota decreased significantly. Notably, *Aquabacterium* sp. has been implicated in skin conditions, such as psoriasis and seborrheic dermatitis, indicating that its reduction may play an important role in the probiotic-assisted treatment of rosacea (67). However, further investigation is warranted to elucidate the specific role of *Aquabacterium* sp. in the progression of rosacea. In the gut microbiota, we identified an enrichment of 14 beneficial microbial taxa, including *V. parvula* and *B. pseudocatenulatum*, alongside a depletion of 10 potential pathogenic taxa, such as *C. citroniae* and *Alistipes finegoldii*. These findings underscore the capacity of probiotics to modulate rosacea-related microbial signatures.

Correlation analysis revealed strong associations between specific gut microbial species. For example, *Gemella haemolysans* showed a strong positive correlation with *Eu.* sp. CAG 274, *S. australis*, and *A.* sp. ICM47. A strong positive correlation was also found between *Eu.* sp. CAG 274 and *B. pseudocatenulatum*, suggesting potential beneficial interactions within the gut microbiota that may contribute to maintaining gut health. Conversely, the negative correlations between *Eu.* sp. CAG 274 and *C. bolteae*, as well as between *C. innocuum* and *Ba. galacturonicus*, may indicate competitive dynamics within the gut microbial ecosystem. Our study also employed the Mantel test to analyze distance matrices of the responsive facial skin and gut microbiota, exploring their potential connections. The results of this analysis suggest the existence of cross-talk between these distinct microbial communities.

Apart from inter- and intra-microbiota connectedness, our study also uncovered significant correlations between the gut microbiota and some rosacea-related physiological indicators. For example, *Ba. vulgatus* showed a positive correlation with the PGA score, indicating that it might aggravate rosacea. Conversely, the probiotic intervention reduced the abundance of *Ba. vulgatus*, potentially ameliorating rosacea symptoms. Notably, *S. parasanguinis*, *Er. ramosum*, and *Cop. cateniformis* exhibited significant negative correlations with facial sebum levels, indicating their potential role in regulating sebum production and inflammation. The increase in these intestinal microorganisms following the probiotic intervention suggests they may contribute to enhancing facial skin health. These findings underscore the complex interactions within the gut microbiota and their diverse effects across different individuals.

While our study revealed significant correlations between gut and facial microbiota and rosacea-related parameters, these associations likely reflect complex systemic interactions rather than direct causality. The “gut–skin axis” theory posits that systemic regulation, such as immune modulation by the gut microbiota, can improve both gut and skin health. Consequently, the changes observed in facial microbiota and skin conditions following probiotic intervention may arise from broader systemic effects rather than a direct influence of gut microbiota on the skin microbiome. This multifactorial perspective is vital for comprehending the gut–skin axis and highlights the need for further research to elucidate the specific pathways involved.

Our findings highlight the inherent complexity and variability in how gut microbiota influences both systemic and localized health outcomes, such as rosacea. The bidirectional interaction between gut microbiota and host immunity adds another layer of complexity, contributing to the variability observed in individual responses to probiotic treatments. For example, our study observed an inverse correlation between the PGA score and levels of IL-8 and LL-37. While these biomarkers are established indicators of inflammation and antimicrobial response, their levels do not consistently align with clinical severity. This discrepancy may suggest that elevated levels reflect compensatory mechanisms or confounding factors related to skin inflammation, rather than serving as direct indicators of rosacea severity. The gut microbiota, alongside factors such as genetics, diet, and environmental influences, collectively affects host physiology, clinical outcomes, and biomarker levels, resulting in personalized responses. Thus, relying solely on IL-8 and LL-37 may not fully capture the complexity of rosacea pathophysiology. This variability underscores the inadequacy of a one-size-fits-all approach, highlighting the need for a multifactorial approach that incorporates additional biomarkers and clinical parameters when evaluating the impact of probiotics on rosacea. Future studies involving larger and more diverse cohorts are essential to better elucidate the mechanisms through which probiotics exert their effects and to optimize therapeutic strategies for rosacea.

Beyond the observed benefits on gut and facial microbiota, skin conditions, and overall inflammation levels, the probiotic intervention notably reduced the abundance of ARGs in the fecal metagenome, particularly those conferring tetracycline resistance. This is particularly noteworthy given that antibiotics like tetracycline are commonly prescribed for rosacea and severe acne. The reduction in ARGs suggests that probiotics may help mitigate the spread of antibiotic resistance, potentially by suppressing microbes that harbor these genes. This highlights the systemic impact of probiotics and their potential utility in addressing broader public health concerns, such as antibiotic resistance, beyond their primary therapeutic target.

Overall, our study provides comprehensive insights into the multifaceted effects of probiotics on gut and facial microbiota, various physiological indicators, and ARGs in the microbiomes in individuals with rosacea. The findings support the potential of probiotics as a promising adjunctive therapy for managing rosacea and enhancing skin health.

## Data Availability

The 16S rRNA and metagenomic sequencing data reported in this paper have been deposited in the Genome Sequence Archive in National Genomics Data, China National Center for Bioinformation / Beijing Institute of Genomics, Chinese Academy of Sciences Centre (accession number: HRA005320; accessible at https://ngdc.cncb.ac.cn/gsa-human).

## AFTER EPUB

*[This article was published on 30 October 2024 with an error in Materials and Methods. The error was corrected on 4 November 2024 and further corrected in the current version, posted on 6 November 2024.]*

## SUPPLEMENTAL MATERIAL

Supplemental Tables msystems.01201-24-s0001.xlsx Tables S1 to S4.

ASM does not own the copyrights to Supplemental Material that may be linked to, or accessed through, an article. The authors have granted ASM a non-exclusive, world-wide license to publish the Supplemental Material files. Please contact the corresponding author directly for reuse.
